# The Effect of Publicly Available COVID-19 Information on the Functioning of Society, Businesses, Government and Local Institutions: A Case Study from Poland

**DOI:** 10.3390/ijerph20032719

**Published:** 2023-02-03

**Authors:** Józef Ober, Janusz Karwot

**Affiliations:** 1 Department of Applied Social Sciences, Faculty of Organization and Management, Silesian University of Technology, Roosevelta 26–28, 41-800 Zabrze, Poland; 2 Sewage and Water Supply Ltd., Pod Lasem 62, 44-210 Rybnik, Poland

**Keywords:** COVID-19, SARS-CoV-2, pandemic, restrictions, quarantine, society, economy

## Abstract

The COVID-19 pandemic has seriously affected many world regions’ societies and economies. The pandemic and the restrictions introduced in response to it have impacted social behaviour and the operation of businesses in various ways. The present study aimed to verify the impact of information on the COVID-19 outbreak in Poland on the frequency with which residents of Rybnik and the surrounding areas chose to stay in their houses or apartments, as well as the functioning of businesses. The originality of the study lies in the use of mediation modelling, in which the following were used as variables describing the epidemic in Poland: new cases of SARS-CoV-2 infection in Poland; the number of deaths caused by SARS-CoV-2 infection in Poland; and the number of people quarantined due to the COVID-19 epidemic in Poland. On the other hand, data on tap water consumption were used as variables describing the frequency with which residents of Rybnik and the surrounding areas chose to stay in their houses or apartments, the operation of businesses in and around the city, and the operation of government and local government institutions. The study’s conclusions confirm that publicly available information about the COVID-19 outbreak in Poland affected the frequency of residents of Rybnik and the surrounding areas staying in their houses or apartments as well as the functioning of businesses in Rybnik and the surrounding areas. The increasing number of people who died due to SARS-CoV-2 infection was associated with the limited functioning of government and local government institutions in and around Rybnik, which contributed to restrictions on the functioning of service industry companies in the city and surrounding areas.

## 1. Introduction

The COVID-19 pandemic has affected the functioning of society and the overall economy of many regions of the world. On 11 March 2020, the World Health Organization (WHO) declared a pandemic state due to the emergence and spread of the SARS-CoV-2 coronavirus causing the COVID-19 Acute Respiratory Infectious Disease 2019 (Coronavirus Disease 2019) [1]. The disease was first identified and described in December 2019 in Wuhan, central China’s Hubei Province, and is believed to have originated in mid–November of the same year [2]. COVID-19 is a highly contagious and dangerous virus that contributes to great devastation and complications in addition to causing illness [3]. SARS-CoV-2 is now the seventh known pathogenic species of human coronavirus and has been classified as a microbial threat. The earlier emergence of the danger in the form of SARS (2002–2003), avian influenza (2003–2006), swine flu (2009–2010), ebola (2013–2016) or measles (2019-present) was characterized by much weaker scope and potency [4]. COVID-19 leads to high mortality rates and causes mass fear and concern. 

The first case of COVID-19 in Poland was reported in March 2020. Since then, the number of patients has been increasing along with short periods of decline. As early as 11 March 2020, there were 274, and by March 2021, more than 1750 people were affected [5]. In January 2022, there were more than 11,000 [5]. By March 2021, more than 115 million SARS-CoV-2 patients had been identified worldwide [5]. By August 2021, there were already 212 million confirmed cases worldwide and more than 4 million confirmed deaths due to COVID-19. The number of patients in each country has changed in waves [5]. The waves of cases in each country fluctuated, and the waves in each region came at different times. 

The pandemic became the basis for many risks, as today no one feels confident about their health, keeping their job, or social position. New mutations of coronavirus, for example, Brazilian, South African, British [4], and omicron are more dangerous than the original Chinese variety. Coronaviruses are currently the most changing viruses due to their high rate of gene substitution: transition or transversion and recombination [6]. The pandemic affects the functioning of the state and society, impacting the state’s condition and citizens’ standard of living [7]. The risk of contracting COVID-19 and its complications was little known at the beginning of the disease’s development. After more than two years of the pandemic, we know a little more about the condition. However, it is still dangerous, and it significantly affects social behaviour. Changes in social behaviour are being studied in many countries, including Australia, France, and Belgium, among others [8]. The risk perceived by the public, regardless of the area, is a subjective assessment made by people in relation to their knowledge and experiences and interacting stimuli in their environment [9]. 

The widespread and global threat of coronavirus has led to an irreversible transformation of the world as we see it, and this is in terms of those aspects that the so-called developed societies took for granted. To curb the spread of the epidemic, the governments of many countries imposed states of emergency, which included many restrictions on movement, public life and the operation of industrial plants and services. The first country to impose a state of emergency, on 17 March, was France, followed by the United States and Poland on 20 March, Germany on 22 March, and the United Kingdom on 23 March [10]. Restrictions introduced by various countries have affected daily life around the world. The implemented limitations varied from country to country [11]. Regulations were necessary to reduce the epidemic’s spread rate and lower the burden on the healthcare system, thus reducing the number of deaths. The increasing number of sick people and deaths contributed to increased anxiety levels in communities and changes in their lifestyles [12]. Communities learned about restrictions and policies to reduce virus transmission from the WHO and the authorities of their countries. This information was conveyed through various types of communication. It was essential to quickly inform the public about the rules of conduct and the danger of the virus [13]. The situation around the pandemic is changing dynamically. Different regions in Poland were very often in the red zone due to an increase in coronavirus infections [14]. 

The COVID-19 pandemic contributed to changes in the job market, as many people were deprived of work. Restaurants, pubs and bars were closed, classes at schools and universities were cancelled, work in specific sectors was moved from in-person to remote at-home offices, and walks in parks and forests became impossible. Many companies closed down due to the restrictions, forcing the dismissal of employees who were only a cost to the enterprise in this situation [15]. The freezing of the economy in the spring of 2020 forced businesses to reduce employees’ working hours and cut wages, which hit the pockets of many families [16]. This has put numerous households in a less-than-optimal financial situation. At the initial stage, incomes did not change in 75.6% of households, and in 22.2% of households, total income decreased [17]. Despite introducing various restrictions, lifestyle changes, and public vaccination, the COVID-19 pandemic is not over, and emerging new virus variants do not bring optimism [18]. Lifestyle modifications are observed in every region of the world. Life has changed regarding physical activity, sedentary lifestyle, sleep and social distance. Lifestyle change has consequences, which have been analyzed in the long and short term [12]. 

The action after the Wuhan outbreak spread taken by the World Health Organization was somewhat late (by several days) and fraught with several controversial decisions. The identification of a new coronavirus species and its rapid spread and relatively high transmissibility led to a series of restrictions in most countries of the world and the WHO’s declaration of a global pandemic [19]. The COVID-19 pandemic contributed to changes in the functioning of entire societies and many areas of the economy. Initial information about the virus and the need to protect against it led governments in many countries to introduce restrictions that affected not only the financial situation of the economy and individual industries but also society itself [20]. 

The COVID-19 pandemic caused a major social crisis and challenged the entire state and social policy system [21]. The social situation, which is heavily influenced by the pandemic and isolation, also places demand on the social welfare system. However, with such a prolonged crisis, this is not the only area that is challenged with properly carrying out the tasks entrusted to it.

Analyzing the literature describing the functioning of the economy during the pandemic and the cause-and-effect relationships that occur with various factors, it can be concluded that there are few examples of linking tap water consumption to the COVID-19 pandemic. At the same time, there is a complete lack of studies that delineate tap water consumption by residents, businesses and government and local government institutions in the context of the pandemic. To fill the identified gap, the authors decided to verify the impact of information on the situation related to the COVID-19 pandemic in Poland on the frequency of stay of residents of Rybnik and the surrounding areas in their houses or apartments and the functioning of businesses. The originality and uniqueness of the study lie in the use of mediation modelling, in which the following variables were used as those describing the epidemic impact in Poland: new cases of SARS-CoV-2 viral infection in Poland; the number of deaths caused by SARS-CoV-2 viral infection in Poland; and the number of people quarantined due to the COVID-19 epidemic. On the other hand, data on tap water consumption were used as variables describing the frequency with which residents of Rybnik and the surrounding areas chose to stay in their houses or apartments, the functioning of businesses in and around this city, and the functioning of government institutions. In doing so, the following research questions were posed:

- Did publicly available information about the COVID-19 outbreak situation in Poland affect the frequency with which residents of Rybnik and the surrounding areas chose to stay in their houses or apartment, and was the operation of businesses indirectly relevant here?
- Did publicly available information about the COVID-19 outbreak situation in Poland affect the functioning of businesses in and around Rybnik, and was the functioning of public institutions indirectly relevant here?

The next part of the article begins with a literature review, which presents the impact of the pandemic on the functioning of the economy and society. It discusses the selected effects of the introduced pandemic restrictions. The following section presents the research methodology and results with a discussion. The paper ends with a summary with conclusions, limitations and opportunities for future research.

## 2. Theoretical Background

In a situation, we face a dynamic social reality factor. A pandemic has caused many changes both socially and economically. The effects of quarantines have been studied more than once, and the results of the studies have been presented in the literature. However, quarantines have not previously been implemented on such a large scale and have not affected so many countries and regions worldwide simultaneously. Quarantines have also not been declared by state authorities for such long periods [22]. A social crisis should be added to the biological features of COVID-19, which spread from human behaviour to individual perceptions of the situation. Based on both previous epidemiological problems and the current one, some conclusions can be drawn about human behaviour. The protective measures in place and the ability to protect oneself from infection are significant. The consequences of viral infection and the risk of developing severe complications have contributed to the growing adherence to government recommendations. 

The rapid spread of COVID-19 and the damage it has caused to the individual’s physical and mental health have been severe. Forced quarantine and confinement as a fight against the disease contributed to restrictions on social mobility and business [23]. The rules put in place contributed to the slowing down of the economy in the first and subsequent periods to economic crises and increasing problems on the part of both business entities and entire national economies [9]. Subsequent quarantines contributed to the deteriorating situation of many financial entities and following changes in social behaviour [24]. Despite the lifting of quarantines, the tourism sector has still not regained its pre-pandemic level of development. The constantly changing situation discouraged tourists from travelling at all [25]. 

The pandemic contributed to the reorganization of family life, which forced many people to relocate. As a result of the pandemic, people often move to rural or suburban areas [22]. One of the reasons for relocating was the need to take care of children and continue working from home. Remote work before the pandemic was rare in many companies. Many entrepreneurs were skeptical about it. After a few months, however, they appreciated its benefits and effects [26]. The pandemic influenced a change in habits and lifestyles [22]. Consumers became more attentive to their health and more cautious in their hygiene to avoid COVID-19 infection, and their eating habits changed, with consumers now often preferring local and homemade foods [1]. In addition to disinfecting hands and washing them more often, wearing masks was mandated, which was meant to prevent airborne transmission of the virus. The requirement to wear masks contributed to a sharp increase in demand for this protective measure [1]. 

COVID-19 and the restrictions introduced in connection with it in Belgium and France contributed to changes in public behaviour. During the initial period of the pandemic, society was quite strict about keeping social distance, more frequent hand washing, or limiting social contact. Sanitary behaviour was, and still is, critical in slowing the spread of infectious diseases. This was particularly important in the initial waves of pandemics when vaccinations and drugs were unavailable [27]. Patterns of social contact and hygiene behaviour are ingrained, making them difficult to modify. 

The blockages resulting from the development of COVID-19 caused many changes in household behaviour in various categories, including water consumption, which decreased significantly in some regions and increased in others. During the pandemic, the population’s remaining at home, in addition to changes in the functioning and organization of daily life, led many households to experience changes in the consumption of primary raw materials such as water, natural gas and electricity. In addition to increased consumption of raw materials by households, an increased amount of solid waste production has been observed due to a reduction in the mobility of the population and an increase in the time spent at home [28]. 

Natural gas is an important raw material the consumption and relevance of which are growing worldwide. During the initial pandemic period from January to June 2020, the global economy reduced gas consumption by 4%. This decrease occurred due to the industry reduction in reserves of this raw material [10]; however, an increase in consumption of it occurred for households. 

Water is a raw material used by households and most market players. Along with electricity, it is one of the primary utilities that households are supplied with [29]. Water is used in various production processes as well as in the provision of services. Water shortage is one of the most significant challenges of modern times, and future generations will have even more problems responding to clean water needs [30]. Households use water in their daily functioning for drinking, food preparation and hygiene, and cleaning. Water is a scarce commodity, and its consumption is being reduced by households and industry which is introducing technologies to its lower consumption [31]. Worldwide, between 35% and 40% of the water we obtain and produce from various sources is wasted. Across Europe, there is no accurate data on the scale of water losses.

For this reason, the new EU Drinking Water Directive mandates that water utilities producing more than 10.000 cubic meters of drinking water or supplying more than 50.000 people per day must measure their water losses [32]. Following the development of the pandemic in southern and eastern England, water consumption has become record high. The global lockdown caused households to change their typical consumption behaviour. Expenditures rose sharply initially, mainly for basic foodstuffs and then for increased water use and other raw materials. Consumer behaviour changes were not just about cutting back on certain expenditures due to the loss of some or all of one’s wages but also due to the inability to bear them or the removal of the need conditioning a particular purchase [33]. In the UK, some regions reported a 35% increase in water consumption during the peak of the lockdown. Likewise, a study in Brazil showed an 11% increase in water consumption. This increase can be attributed to people staying indoors and an increase in preventive behaviours such as hand washing. The water demand in England and Wales households increased by 55% [34]. Studies in many countries have shown that changing habits in Australia, for example, regarding more frequent hand washing, may have significantly impacted higher water consumption. The use of appropriate washing products was considered to reduce the personal risk of contracting COVID-19 [8]. Increased household water consumption was observed in southern Brazil. A decrease in water demand in industry and public institutions has been reported in this region [35]. Changes in water demand have been studied relatively well using data from Austin, Texas. Data collected by this entity show that during periods of lockdown, an increase in water demand is observed, and during periods of opening, the demand decreases [35]. 

Changes in water demand have forced adjustments in water companies’ approach to water requirements. Increased water demand in many regions has significantly impacted water quality. Water demand depends on geographic area and weekday patterns [35]. Water quality is a problem in many parts of the world, including Poland. In Poland, water quality requirements are determined by current regulations, including standards imposed by the EU [36]. Reasonable satisfaction of residents’ water needs requires appropriate infrastructure, which should meet the increasing water needs of residents. The water system should comply with sanitary and hygienic standards [37]. 

The amount of water supplied to a property is determined based on the readings of the main water meter. A decrease in water consumption in Poland has been observed over the years. This is related, among other things, to the change in billing for water consumption from a flat fee to water–meter readings. Individual monitoring of the amount of water consumption in pre-pandemic households decreased consumption by an average of 15–25% [38]. A decrease in water consumption means, at the same time, a quantitative reduction in water sales by water companies and, therefore, an increase in fixed costs with a limited rise in variable costs, that is, the unit price per m³ of water [39]. 

During the pandemic, when most of life moved into homes, one would expect an increase in water consumption, like that of gas and electricity. However, the trend was also the opposite. For example, a decrease in water consumption was reported in 2020 compared to 2019 in southern Italy [35]. 

Successful management of a pandemic depends on understanding public perceptions and behaviours, including fears, frequency of personal hygiene use, and gaining immunity by getting vaccinated or surviving the infection [13]. Countries worldwide have sought to raise public awareness of the virus, as well as conducted research on changes in social behaviour and lifestyle. Studies performed to date on the impact of population confinement on their behaviour suggest that too long a period of closure reduces the effectiveness of implemented interventions [28]. The pandemic has shocked the world and has highlighted areas where changes need to be made. It showed the weaknesses of many sectors, including healthcare [40]. It also showed the urgent need to implement environmentally sustainable behaviours and actions that play an essential role in human life [41,42,43,44].

## 3. Materials and Methods

### 3.1. Purpose of the Study

The present study was designed to verify the impact of information on the situation related to the COVID-19 epidemic in Poland on the frequency with which residents of Rybnik and the surrounding areas chose to stay in their houses or apartments, as well as the functioning of businesses. In doing so, the following research questions were posed:

- Did publicly available information about the COVID-19 outbreak situation in Poland affect the frequency with which residents of Rybnik and the surrounding areas chose to stay in their houses or apartments, and was the functioning of businesses indirectly relevant here?
- Did publicly available information about the situation related to the COVID-19 epidemic in Poland affect the functioning of businesses in Rybnik and the surrounding areas, and was the functioning of public institutions of indirect relevance here?

Due to the nature of the research and the selection of variables, a case study research strategy was used, which involves an in-depth exploration of events, activities, processes and/or people [45]. This research approach identifies concepts that can be replicated and potential errors that can be avoided. Additionally, it requires a great deal of time and effort, and different procedures are used to gather detailed information over a more extended period [46].

### 3.2. Research Tool

The empirical material was collected based on data available on the web portal [47] on the COVID-19 outbreak and data from the Water Supply and Sewerage Company Ltd. in Rybnik (PWiK Rybnik) on tap water consumption (in m³) in and around Rybnik. The data mentioned above concerned monthly summaries from March 2020 to July 2021 and were collected for this study. Rybnik is a city with county rights, located in southern Poland on an area of 148 square kilometres divided into 27 districts with a population of approximately 140,000 [48]. Rybnik ranks sixteenth on the list of Poland’s most significant cities by area and twenty-fifth by people [49].

### 3.3. The Subject of Statistical Analysis

The impact of information on the COVID-19 epidemic situation in Poland on the frequency of stay of residents of Rybnik and the surrounding areas in their houses or apartments, as well as the operation of businesses in the city and the surrounding area, was the main focus of statistical analysis. The variables (information) describing the epidemic in Poland were defined as follows:

- The number of new cases of SARS-CoV-2 viral infection (in thousands) in Poland (“New Cases”);
- The number of deaths caused by SARS-CoV-2 viral infection in Poland (“Deaths”);
- The number of people quarantined (in thousands) due to the COVID-19 outbreak (“Quarantine”).

On the other hand, data on tap water consumption (in thousands of cubic meters) were used as variables describing the frequency with which residents of Rybnik and the surrounding areas chose to stay in their houses or apartments, the functioning of businesses in and around the city, and the functioning of government institutions. In doing so, it was assumed that tap water in the era of the pandemic (due to recommendations for frequent hand washing) is necessary for the functioning of both people staying in their own homes and employees of enterprises and government and local government institutions, regardless of the type of activity and tasks performed. Hence, changes in tap water consumption may be a reflection of the frequency with which residents of Rybnik and the surrounding areas chose to stay in their own houses or apartments (more water consumption means higher frequency of staying at home), as well as the functioning of enterprises and institution (lesser water consumption means a reduction in activities or a shift to remote work). The above data is applied to the following entities:

- Households, including residential buildings, collective housing, and dwellings;
- The business sector, including industry, commerce and services;
- Government and local government institutions, including schools, kindergartens and other government and local government institutions.

Statistical analysis was aimed at assessing the impact of the above information regarding the epidemic situation in Poland on the behaviour of residents of Rybnik and its surroundings in terms of staying in their houses or apartments, as well as learning about the possible consequences of the nationwide epidemic for businesses in the city and its surroundings. An essential aim of the above research was to learn about the mediating role of the functioning of businesses in Rybnik and the surrounding areas in the relationship between the epidemic in Poland and the staying of residents of Rybnik and the surrounding areas in their houses or apartments and to learn about the mediating role of the functioning of public institutions in Rybnik and the surrounding areas in the relationship between the above situation and the functioning of businesses in this city and the surrounding area.

### 3.4. Methodology of Statistical Analysis

**Figure 1:**
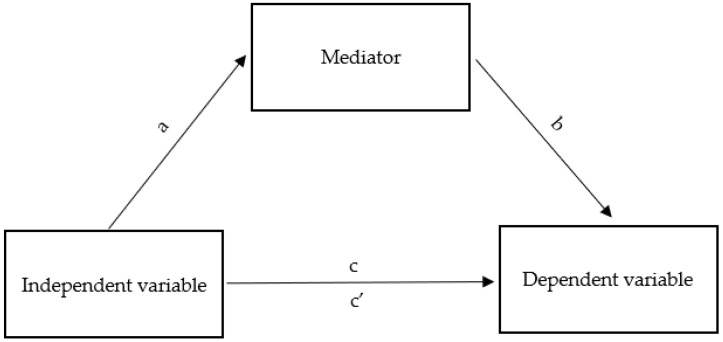
Relationship diagram in the mediation model.

The material collected during the study was subjected to quantitative analysis. Due to their measurable nature, the results of the analyzed parameters were presented using the values of basic descriptive statistics. The Kolmogorov–Smirnov test verified the distribution of quantitative variables. To build a mediation model, mediation analysis in the regression was applied, using the classical approach of Baron and Kenny [50], which consists of testing the mediation relationship by checking other relationships in three steps (Figure 1):

- independent variable with the dependent variable (direct dependence; path c),
- independent variable with a mediator (path a) and mediator with the dependent variable (path b),
- independent variable with the dependent variable (direct dependence) when both the independent variable and the mediator are included in the model (path c′) [50].

Mediation is complete when the independent variable ceases to significantly predict the dependent variable in a regression model that includes a mediator. Mediation is partial if the effect of the independent variable on the dependent variable only weakens [51]. In the case of partial mediation, the Goodman test reporting the significance of the product of the regression coefficients for path a and path b after the introduction of a mediator, recommended in the situation of analyses on small samples (less than 50 observations), was used as a supplement to the analyses.

The results were statistically analyzed using Statistica v.13.3 PL Tulsa, OK, USA. Analysis by the Goodman test was performed using a calculator available on the website [52]. Significant test probability was taken as *p* < 0.05.

## 4. Results and Discussion

### 4.1. Analysis of Total Results—Variables Included in the Research Model

**Figure 2:**
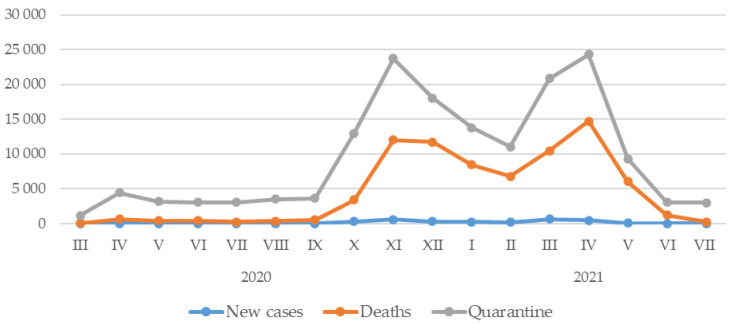
Data on the situation in Poland in relation to the COVID-19 epidemic by month of the study period.

The discussion of the results of this study began with an analysis of descriptive statistics and the distribution of outcomes by period. In the case of data for the COVID-19 outbreak in Poland, an increase in new cases of SARS-CoV-2 infection, deaths due to the virus, as well as people quarantined after September 2020 was observed. This increase continued for two months, followed by a decrease in the aforementioned indicators, until February 2021. For the following months, there was again an increase in the aforementioned indicators until April 2021, and from then until July 2021, the values of the aforementioned indicators decreased (Figure 2). Throughout the period from March 2020 to July 2021, the average monthly number of new cases of SARS-CoV-2 viral infection was 167.61 (SD = 217.14; 2.31–644.09) thousand, while the number of deaths was an average of 4394.88 (SD = 4942.76; 33–14,250) per month. On the other hand, during quarantine, the average was 4977.09 (SD = 3308.71; 1094.3–11,679.16) thousand people per month (Table 1).

**Figure 3:**
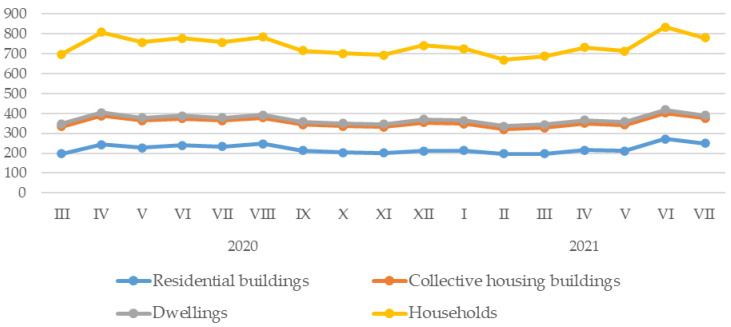
Household water consumption in Rybnik and the surrounding areas by month of the study period.

Tap water consumption in household buildings in Rybnik and the surrounding areas was distributed at a similar level throughout the studied period of the COVID-19 pandemic, while slight increases in water consumption were observed in the months of April and August 2020, at the end of 2020–2021, and in April and June 2021. This was true for residential buildings, collective housing, dwellings and all households (Figure 3). Among all these groups of tap water users, residential buildings displayed the highest consumption rates, averaging 221.73 (SD = 22.1; 196.46–271.92) thousand cubic meters per month. Second in this respect were collective housing buildings, where tap water consumption averaged 133.01 (SD = 5.07; 123.7–144) thousand cubic meters per month. Significantly lower tap water consumption was in dwellings, averaging 15.09 (SD = 0.57; 14.45–16.57) thousand cubic meters per month. In contrast, all households consumed an average of 369.82 (SD = 22.97; 334.92–417.28) thousand cubic meters of tap water per month in the analyzed period (Table 2).

The study area is conventionally divided into 27 zones in the SCADA system, where water consumption data, including increases and decreases, were analyzed on an ongoing basis. Such daily analysis allowed the network’s operating parameters to be set appropriately so there would be no consumer nuisance in water supply and wastewater collection.

**Figure 4:**
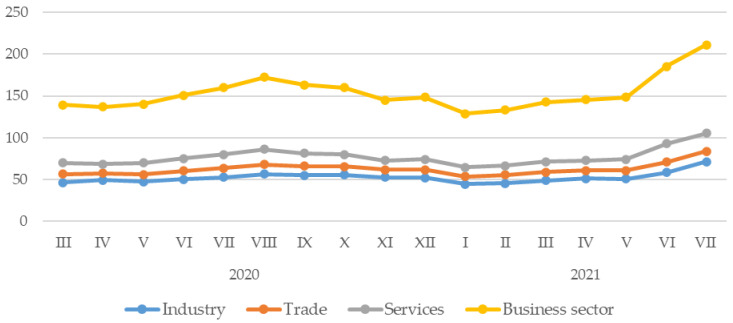
Water consumption in the business sector in Rybnik and the surrounding area by month of the study period.

As for the business sector in Rybnik and the surrounding areas, there was a gradual increase in tap water consumption (for both industries, trade and services, and the overall business sector in general) from April to August 2020. A gradual decline in water consumption followed this month until January 2021 (with a slight deviation from this downward trend in December 2020). After that, a gradual increase in water consumption by companies is noticeable, with the growth taking a more pronounced form after May (Figure 4). On the other hand, over the entire period analyzed, the average tap water consumption in the business sector was 76.77 (SD = 10.36; 64.39–105.54) thousand cubic meters per month. Companies in the industrial sector consumed an average of 52.29 (SD = 6.24; 44.64–71.18) thousand cubic meters of water per month, the trade sector consumed 10.11 (SD = 1.15; 8.13–12.45) thousand cubic meters, and the service sector consumed 14.37 (SD = 3.55; 10.58–22.08) thousand cubic meters, respectively (Table 3).

**Figure 5:**
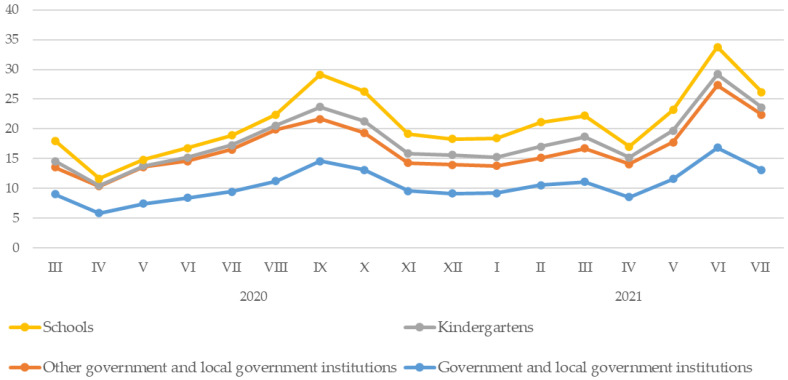
Water consumption in government institutions in Rybnik and the surrounding areas by month of the period under review.

Government and local government institutions recorded the most significant jumps in all groups’ tap water consumption. After the first month of the analyzed period (in April 2020), there was a marked decline in tap water consumption by schools, kindergartens, other government and local government institutions, and all such entities in general. In the next five months, until September, there was a gradual increase in the aforementioned indicator, followed by a decrease in the next two months, to remain stable for the period from November 2020 to January 2021. After that, water consumption increased until July 2021 (except for April, when lockdown tightening was introduced nationwide) (Figure 5). Over this period, the average monthly tap water consumption in all government institutions in Rybnik and the surrounding neighbourhoods was 10.52 (SD = 2.74; 5.84–16.88) thousand cubic meters. Schools consumed an average of 2.97 (SD = 1.34; 1.04–5.44) thousand cubic meters of water per month, kindergartens consumed 1.3 (SD = 0.64; 0.13–2.02) thousand cubic meters, and other government institutions consumed 6.25 (SD = 1.79; 4.54–10.45) thousand cubic meters of water per month (Table 4).

It should be noted that the data acquired was subject to daily changes at different times. Services that could be performed remotely moved to the Internet, stores operated in different modes and, for example, seniors could make purchases only during certain morning hours. Employees of critical infrastructure companies and such, in addition to water supply, gas and electricity supply, obtained knowledge of the degree of “household population” from data correlated with information systems (e.g., SCADA). At the height of the pandemic, it was possible to see a high rate of change in water consumption. Still, the accuracy of the assumptions made it possible to deliver water continuously.

In order to select appropriate variables for the construction of the mediation model using multiple linear regression analysis, Pearson’s linear correlation coefficients were verified between the independent variables (concerning the situation in Poland in relation to the COVID-19 epidemic) and the dependent variables and acting as a mediator in the model (concerning tap water consumption in households, the business sector and government institutions in and around Rybnik). It was determined that there was a statistically significant negative correlation between all independent variables (i.e., “New Cases,” “Deaths”, and “Quarantine”) and water consumption in residential buildings, total households and businesses in the service industry. In addition, there was a statistically significant negative association between the number of COVID-19 deaths and water consumption in other government and local government institutions. All of the above relationships were of moderate strength. The remaining tap water consumption variables were not significantly associated with any epidemic variables (Table 5).

Based on these results, all variables describing the situation in Poland in relation to the COVID-19 epidemic (i.e., “New cases,” “Deaths”, and “Quarantine”) were selected for the mediation model. Tap water consumption in residential buildings (“Residential buildings”) and all households in general (“Households”) were used as variables describing the stay of residents of Rybnik and the surrounding areas in their houses or apartments. In the case of businesses, only the results for tap water consumption of companies in the service industry were related to the COVID-19 variables. Hence, only this variable (“Services”) was taken as describing the operation of businesses in Rybnik and the surrounding areas. On the other hand, in the case of government institutions, only the water consumption data of other public institutions were statistically significantly correlated with any of the variables describing the epidemic in Poland, so, as in the case of the business sector, only this variable (“Other government and local government institutions”) was included in the model as describing the functioning of the aforementioned institutions in and around Rybnik.

### 4.2. COVID-19 and Staying at Home—The Mediating Role of Corporate Functioning

**Figure 6:**
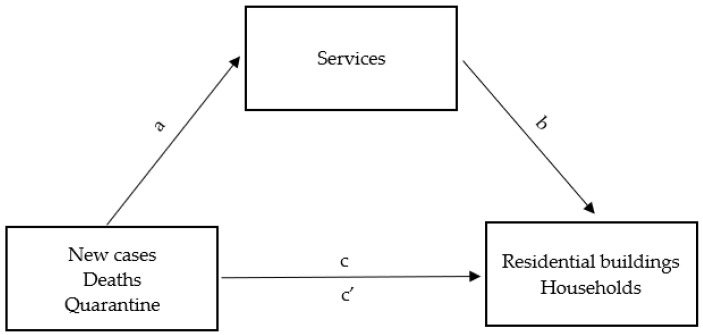
Mediation diagram used in the study of the mediating role of business operation in Rybnik and its surroundings in the relationship between the situation in Poland concerning the COVID-19 epidemic and the choice of the residents of Rybnik and its surroundings to stay in households.

Next, a mediation model was constructed, assuming the existence of a relationship between the epidemic in Poland and the choice of residents of Rybnik and its surroundings to stay in their houses or apartments, taking into account the mediating role of the operation of service industry enterprises in this city and its surroundings. A diagram of the relationship between the aforementioned variables in the mediation model is presented in Figure 6.

#### 4.2.1. New Cases of SARS-CoV-2 Infection in Poland

The mediation analysis conducted in the first step confirmed the direct correlation between the stay of residents of Rybnik and the surrounding areas in their houses or apartments with information on new cases of SARS-CoV-2 viral infection in Poland. The developed regression model proved to be a good fit and showed that the more recent cases of COVID–19 infection were reported, the lower the consumption of tap water in residential buildings (β = −0.59; *p* < 0.05) and households in general (β = −0.58; *p* < 0.05) was observed. In the second step, the relationship between the independent variable of new cases of COVID-19 and the mediator defining the performance of enterprises in the service industry in and around Rybnik was tested. It turned out that the above relationship was also statistically significant (β = −0.53; *p* < 0.05), and the whole model was well fitted to the data. In this case, it was shown that a higher number of new cases of SARS-CoV-2 viral infection was also associated with lower tap water consumption in service industry enterprises in and around Rybnik.

**Figure 7:**
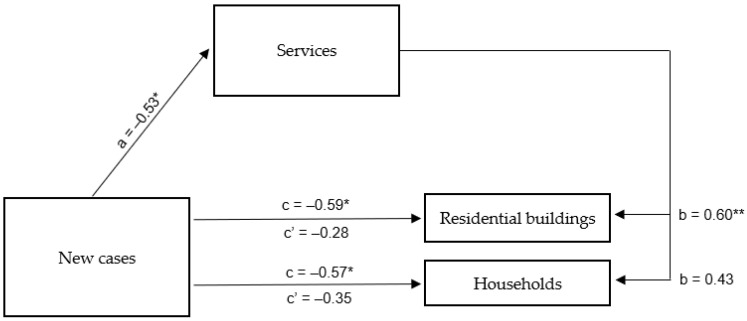
Mediation model for the effect of new cases of SARS-CoV-2 viral infection in Poland on the stay in households of residents of Rybnik and the surrounding areas—the mediating role of the functioning of the service industry in Rybnik and the surrounding areas. * *p* < 0.05; ** *p* < 0.01.

On the other hand, in the third step, taking into account both the mediator and the independent variable, the role of the latter (i.e., the number of new cases of SARS-CoV-2 viral infection) in water consumption decreased and proved statistically insignificant, both for residential buildings (β = −0.28; *p* = 0.179) and households in general (β = –0.35; *p* = 0.153). In the case of residential buildings, the mediator was strongly related to the dependent variable (β = 0.60; *p* < 0.01), while in the cases of total households, the relationship with the mediator was statistically insignificant (β = 0.43; *p* < 0.08) (Table 6). A summary of the obtained β coefficients is shown in Figure 7.

The above results indicate that the operation of the service industry in Rybnik and the surrounding areas completely mediates the relationship between information about new cases of COVID-19 infection and the frequency with which residents of Rybnik and the surrounding areas chose to stay in their houses or apartments, as determined by residential tap water consumption. The higher number of new cases of SARS-CoV-2 viral infection in Poland favoured restrictions on the operation of service industry companies in and around Rybnik, which in turn led residents of that city and its surroundings to less frequently choose to stay in residential buildings.

#### 4.2.2. Deaths Due to SARS-CoV-2 Viral Infection in Poland

**Figure 8:**
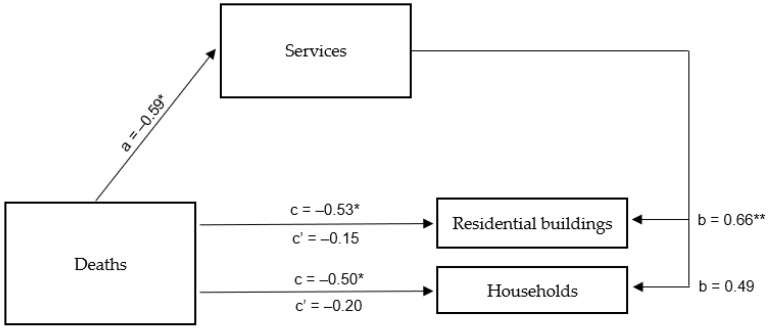
Mediation model for the impact of deaths due to SARS-CoV-2 viral infection in Poland on the choice of residents of Rybnik and surrounding areas to stay in households —mediating role of service industry functioning in Rybnik and surrounding areas. * *p* < 0.05; ** *p* < 0.01.

The mediation analysis showed a direct relationship between the residence of inhabitants of Rybnik and the surrounding area in their homes or apartments and information on the number of COVID-19-related deaths in Poland. The regression model developed for the aforementioned relationship was a good fit. It was shown that a higher number of deaths due to SARS-CoV-2 infection was associated with lower tap water consumption in both residential buildings (β = −0.53; *p* < 0.05) and households in general (β = −0.50; *p* < 0.05). The relationship between information on the aforementioned deaths and a mediator in the form of the operation of service industry enterprises in and around Rybnik was also determined to be statistically significant (β = −0.59; *p* < 0.05), and the overall model fit the data well. On the other hand, when the mediator and independent variable were considered simultaneously, it turned out that the role of the latter in tap water consumption by residents of Rybnik and the surrounding areas decreased and did not reach statistical significance, both for residential buildings (β = −0.15; *p* = 0.511) and households in general (β = −0.20; *p* = 0. 436). In the relationship with water consumption in residential buildings, the mediator was strongly associated with the dependent variable (β = 0.66; *p* < 0.01), while in the relationship with water consumption in all households in general, the association with the mediator proved to be statistically insignificant (β = 0.49; *p* < 0.073) (Table 7). A summary of the obtained β coefficients is shown in Figure 8.

The obtained results showed that, as in the case of information about new issues of SARS-CoV-2 viral infection, the functioning of the service industry in Rybnik and its surroundings completely mediates the relationship between knowledge about deaths caused by the aforementioned infection and the frequency with which residents of Rybnik and its surroundings chose to stay in their houses or apartments, determined by the consumption of tap water in residential buildings. The more people died from COVID-19 infection, the greater the restrictions on the operation of service industry companies in and around Rybnik and, consequently, the less frequent the choice to stay in residential buildings by residents of this city and surrounding areas.

#### 4.2.3. People Quarantined in Connection with COVID-19 Outbreak in Poland

**Figure 9:**
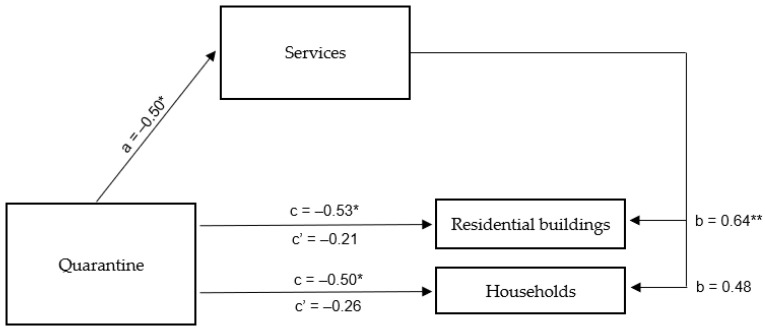
Mediation model for the effect of the number of people quarantined due to the COVID-19 outbreak in Poland on the choice of residents of Rybnik and the surrounding areas to stay in households —the mediating role of the operation of the service industry in Rybnik and the surrounding areas. * *p* < 0.05; ** *p* < 0.01.

The results of the mediation analysis of the relationship between the number of people quarantined due to the COVID-19 epidemic in Poland and the choice of residents of Rybnik and the surrounding areas to stay in their households with the mediating role of the operation of the service industry in this city and the surrounding areas showed similar relationships between the variables as was the case with information on new cases of infection and deaths. As a first step, the above analysis confirmed a direct connection between the residents’ of Rybnik and the surrounding areas choice to stay in their houses or apartments and information on the number of people quarantined due to the COVID-19 epidemic in Poland. The more people were quarantined, the less tap water the residents of Rybnik and the surrounding area used in residential buildings (β = −0.53; *p* < 0.05) and households in general (β = –0.50; *p* < 0.05), and the developed regression model was a good fit. In the next step, there a statistically significant negative relationship was also observed between the independent variable and the mediator (β = −0.50; *p* < 0.05). Information about a more substantial number of quarantined people favoured lower tap water consumption in service industry companies in and around Rybnik. Finally, tests of the relationship between the independent variable and the dependent variable, taking into account the mediator in the form of the operation of the service industry (tap water consumption by companies in this industry), showed that the role of the number of quarantined people decreased and became statistically insignificant for both residential buildings (β = −0.21; *p* = 0.302) and total households (β = −0.26; *p* = 0.282). At the same time, in the case of residential buildings, the mediator was strongly related to the dependent variable (β = 0.64; *p* < 0.01), while in the case of total households, the relationship with the mediator did not reach statistical significance (β = 0.48; *p* < 0.057) (Table 8). A summary of the obtained β coefficients is shown in Figure 9.

Summing up, the mediation analysis showed that, as in the case of the other two independent variables concerning the epidemic in Poland, the operation of the service industry in Rybnik and its surroundings completely mediates the relationship between information on the number of people quarantined due to the COVID-19 epidemic and the frequency with which residents of Rybnik and its surrounding areas chose to stay in their houses or apartments, as determined by the consumption of tap water in residential buildings. The more people quarantined nationwide, the greater the restrictions on the operation of service industry companies in and around Rybnik, which led to a less frequent stay in residential buildings by residents of this city and surrounding areas.

The results of a study conducted in India [53] show an increase of several per cent in water consumption along with electricity consumption and expenses during the COVID-19 pandemic. The authors of this study indicate an increase in the frequency of daily hand washing from 6 to 11–16 depending on the region and degree of urbanization. These studies also reported a 30% to 50% (depending on urbanization) increase in the frequency of bathing from once to twice a day during the pandemic. Another element was washing clothes, where the increase observed was approximately 10%. Similar studies were also conducted in Brazil [54], where survey respondents indicated more frequent hand washing and bathing. These studies do not confirm those undertaken in Rybnik and the surrounding areas, where, conversely, residents decreased water consumption during the pandemic period. However, water consumption data in India and Brazil were calculated based on the declarations of respondents filling out questionnaires. In contrast, the studies in Rybnik and its surroundings were based on actual water consumption as indicated by water meters. It can be assumed that the reported reduction in household water consumption during the pandemic was related to changes in behaviour regarding the frequency of bathing and washing and was due to intentional savings associated with the national and global emergency.

Studies in the United States [55] indicate that water consumption during the pandemic in residential buildings did not change in low-income groups, even during the total lockdown, while overall, average water consumption by all consumers (businesses and apartments) increased from 11.80 to 13.5%, which is not the same as the data in the study conducted in and around Rybnik. The result may be that more companies in and around the Rybnik area have closed and downsized compared to the study area in the United States.

### 4.3. COVID-19 and the Functioning of Businesses—The Mediating Role of the Functioning of Government and Local Government Institutions

**Figure 10:**
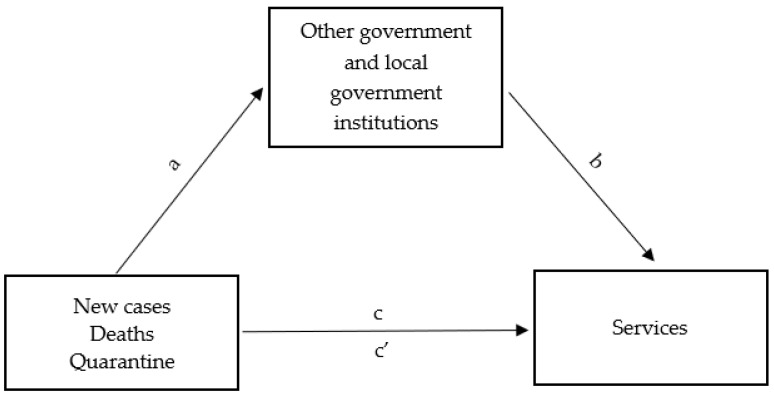
Mediation diagram used in the study of the mediating role of the functioning of government institutions in Rybnik and its surrounding areas in the relationship between the situation in Poland concerning the COVID-19 epidemic and the functioning of businesses in Rybnik and its surrounding areas.

Subsequently, a mediation model was developed, assuming the existence of a relationship between the epidemic in Poland and the functioning of enterprises in Rybnik and its surroundings, taking into account the mediating role of the functioning of local government institutions in the city and its surroundings. The diagram of the relationship between the aforementioned variables in the mediation model is presented in Figure 10.

#### 4.3.1. New Cases of SARS-CoV-2 Infection in Poland

**Figure 11:**
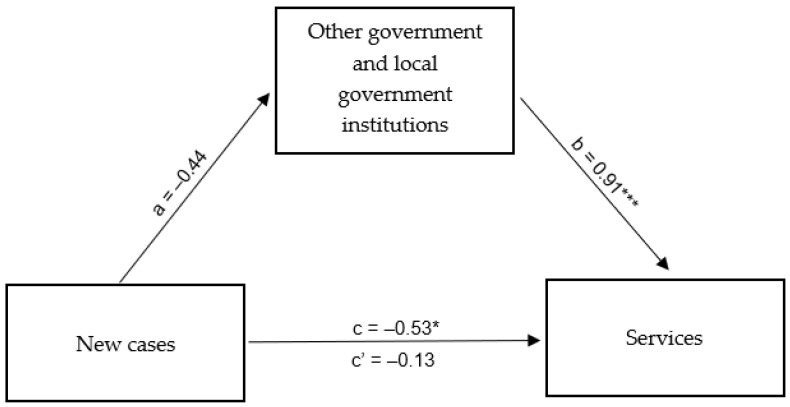
Mediation model for the effect of new cases of SARS-CoV-2 infection in Poland on the functioning of the service industry in Rybnik and its surrounding areas—mediation role of the functioning of other government and local government institutions in Rybnik and its surrounding areas. * *p* < 0.05; *** *p* < 0.001.

Based on the mediation analysis results, a direct relationship was confirmed between the operation of service industry companies in and around Rybnik and information on the number of new cases of SARS-CoV-2 viral infection in Poland. The developed regression model was a good fit and showed that the relationship between the information mentioned above on the epidemic in Poland and tap water consumption in service companies was statistically significant and negative (β = −0.53; *p* < 0.05): the more new cases of COVID-19 infection reported in Poland, the lower the tap water consumption in the companies in Rybnik mentioned above and the surrounding areas. In the next step, testing the direct relationship between the independent variable (“New cases”) and the mediator in the form of the functioning of public institutions (“Other government and local government institutions”) showed that the relationship was also negative, but did not reach statistical significance (β = −0.44; *p* < 0.08). This means that the functioning of the aforementioned institutions (defined by the consumption of tap water by other government and local government institutions) was not a mediator of the relationship between new cases of SARS-CoV-2 viral infection in Poland and the functioning of the service industry in Rybnik and its surrounding areas—despite the very strong mediator relationship with the dependent variable (β = 0.91; *p* < 0.001) and the decreasing role of the independent variable at the same time (β = −0.13; *p* < 0.076) (Table 9). A summary of the obtained β coefficients is shown in Figure 11.

#### 4.3.2. Deaths Caused by SARS-CoV-2 Infection in Poland

**Figure 12:**
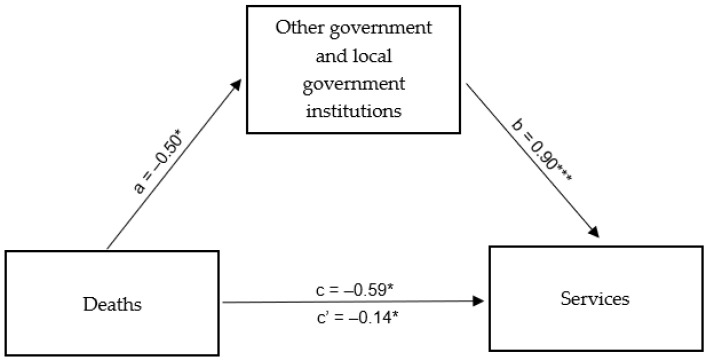
Mediation model for the effect of deaths caused by SARS-CoV-2 infection in Poland on the functioning of the service industry in Rybnik and the surrounding areas—the mediating role of the functioning of other government and local government institutions in Rybnik and the surrounding areas. * *p* < 0.05; *** *p* < 0.001.

In the course of developing a mediation model for the effect of the number of deaths due to SARS-CoV-2 viral infection in Poland on the functioning of the service industry in Rybnik and its surrounding areas with the mediating role of the functioning of government institutions in this city and its surrounding areas, the existence of a direct relationship between the aforementioned information on the epidemic and the consumption of tap water by companies in the service industry in this city and its surrounding areas was confirmed in the first step. The relationship mentioned above was determined to be statistically significant and negative (β = −0.59; *p* < 0.05): the more COVID-19 deaths were reported, the lower the tap water consumption of the aforementioned companies in and around Rybnik was observed, and the whole model was a good fit. In the second step, a direct relationship was tested between the aforementioned independent variable and the mediator, which was the functioning of other government and local government institutions in and around Rybnik. In this case, the developed regression model was also a good fit and revealed a statistically significant and negative relationship between the aforementioned variables (β = −0.50; *p* < 0.05). Finally, in the model considering both the mediator and the independent variable, the role of the independent variable in tap water consumption by service industry enterprises in and around Rybnik decreased while remaining statistically significant (β = −0.14; *p* < 0.05) and the mediator was very strongly related to the dependent variable (β = 0.90; *p* < 0.001) (Table 10). A summary of the obtained β coefficients is shown in Figure 12.

The result indicating partial mediation of the functioning of other government and local government institutions in and around Rybnik was confirmed by the Goodman test, which was determined to be statistically significant: Z = 2.12; *p* < 0.05. Information on subsequent deaths caused by SARS-CoV-2 viral infection in Poland contributed significantly to the introduction of restrictions on the functioning of public institutions in and around Rybnik, which in turn led to restrictions on service companies’ activities in the city and its surrounding areas.

#### 4.3.3. People Quarantined in Connection with COVID-19 Outbreak in Poland

**Figure 13:**
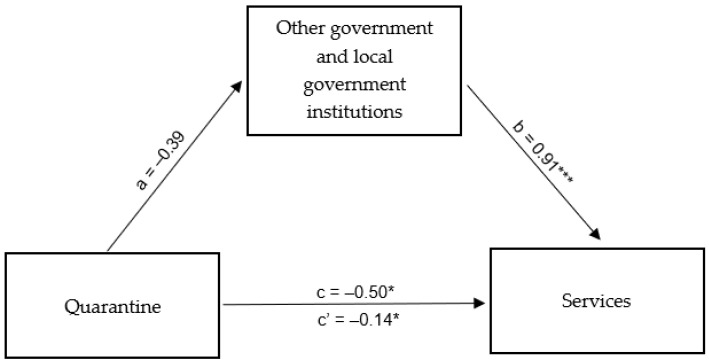
Mediation model for the effect of the number of people quarantined due to the COVID-19 epidemic in Poland on the functioning of the service industry in Rybnik and the surrounding areas—the mediating role of the functioning of other government and local government institutions in Rybnik and the surrounding areas. * *p* < 0.05; *** *p* < 0.001.

As a first step, the mediation analysis confirmed a direct relationship between the operation of service industry enterprises in and around Rybnik and information on the number of people quarantined due to the COVID-19 epidemic in Poland. The model proved to be a good fit and indicated a statistically significant negative relationship between the aforementioned variables (β = −0.53; *p* < 0.05): a higher number of quarantined people was associated with lower tap water consumption in service companies operating in and around Rybnik. In the next step, the direct relationship between the aforementioned independent variable and the mediator determined by the functioning of other government institutions in Rybnik and the surrounding areas was tested. It turned out that water consumption by the mentioned institutions was not significantly related to the number of quarantined people (β = −0.39; *p* = 0.118). Thus, the functioning of the institutions was not a mediator of the relationship between the number of people in quarantine and the functioning of the service industry in and around Rybnik, although there was a strong mediator relationship with the dependent variable (β = 0.91; *p* < 0.001) while at the same time, the role of the independent variable was reduced (β = −0.14; *p* < 0.05) (Table 11). A summary of the obtained β coefficients is shown in Figure 13.

Studies conducted in Poland [56,57], South Korea [58] and other pandemic-affected countries [59], among others, indicate that public information on the number of illnesses, quarantines and deaths due to COVID-19 has an impact on reducing activity, especially of service companies, which confirms the results of studies from Rybnik and the surrounding areas. In contrast, a study from the UK [34] shows an increase in water consumption by businesses during the pandemic, while water consumption by households remains at similar levels. The authors of these studies note a significant change in the hours of water consumption by household residents, indicating a substantial increase from morning to afternoon hours. This can be explained by the fact that residents who remain in their apartments throughout the day change their habits regarding the hours of bathing, washing or doing the dishes. Although the study in the area of Rybnik and its surroundings did not consider the exact hours of water consumption for this manuscript, these observations can be confirmed.

### 4.4. Summary of the Model of the Mediating Role of Mediators between Independent Variables and Dependent Variables

The higher number of new cases of SARS-CoV-2 viral infection, quarantined persons and deaths across the country, and thus the increased epidemic state in Poland, was associated with more significant restrictions on the operation of service companies in Rybnik and, as a consequence, led to residents of this city less frequently choosing to stay in residential buildings and being more economical in their use of tap water in their homes and apartments. In addition, information about further deaths caused by SARS-CoV-2 viral infection in Poland led to restrictions on the operation of public institutions in Rybnik, which partly led to restrictions on the operations of service companies in that city as well (Table 12).

The analyses cited and the assumptions made for the operation of critical infrastructure are one of the first attempts to systematize the optimal process of the water supply network under the emergence of an unknown threat. Based on this example, it is possible to draw conclusions and create crisis scenarios allowing one to survive a challenging period in a relatively stable way.

## 5. Conclusions

Publicly available information about the COVID-19 epidemic in Poland influenced the frequency with which residents of Rybnik and the surrounding areas chose to stay in their houses or apartments (determined by the tap water consumed), and the operation of businesses was of indirect importance here. The worsening epidemiological situation (i.e., the increasing number of new cases of SARS-CoV-2 viral infection, the growing number of deaths from it, and the rising number of people under quarantine) has made it necessary to reduce the scale of operations of service companies in Rybnik and the surrounding areas (reducing tap water consumption).

Publicly available information on the situation related to the COVID-19 epidemic in Poland affected the functioning of businesses in and around Rybnik, with the functioning of public institutions being of indirect importance only in the case of information on deaths caused by COVID-19 infection. The increasing number of people who died due to SARS-CoV-2 disease was associated with the reduced functioning of government institutions in and around Rybnik, which contributed to restrictions on the functioning of service industry companies in and around this city.

In addition, from a practical point of view, the acquisition and deep analysis of data allowed the management of PWiK Rybnik to draw conclusions on an ongoing basis and react to the formula for water supply and wastewater collection so that the entire process runs smoothly. This course of action made it possible to develop good practices to control water pressure in areas of increased water intake because city residents remain in their homes.

One of the most important recommendations for a medium-sized city is to be cautious in decision-making regarding water and sewage network control during the pandemic and to monitor water’s physical and chemical parameters on an ongoing basis due to their instability. This is conditioned by the increased rates of people falling ill and being quarantined, making it necessary to complete daily parameterizations of the water supply network operation.

It should be borne in mind that the results of this study have some limitations. The first is related to the small sample size due to the restricted and seasonal duration of the COVID-19 pandemic in Poland. The second limitation is associated with the impossibility of assessing the motivation of individuals to change their use of tap water, mainly private persons. The development of the pandemic, the restrictions introduced, and the consequent constraints on the operation of companies in the service industry (which were most adversely affected by the epidemic) may have created a sense of uncertainty among the residents of Rybnik and the surrounding areas about further developments and motivated them to save on their bills (including tap water), regardless of the issue of staying in their houses or apartments. Depending on the further development of the situation related to the pandemic, opportunities for further research and monitoring of the parameters in question throughout the country and/or similar analyses in other countries for comparison purposes could be identified. 

## Figures and Tables

**Table 1:** Data on the situation in Poland in relation to the COVID-19 epidemic (descriptive statistics).

	Descriptive Statistics					
	Mean ± Stand. Dev.	Median (Q25–Q75)	Min.–Max.	Confidence Interval		Stand. Error
				−95.00%	+95.00%	
New cases (in thousands)	167.61 ± 217.14	24.14 (10.61–271.22)	2.31–644.09	55.97	279.25	52.66
Deaths	4394.88 ± 4942.76	1188 (400–8258)	33–14,250	1853.55	6936.22	1198.80
Quarantine (in thousands)	4977.09 ± 3308.71	3298.26 (2776.1–6343.66)	1094.3–11,679.16	3275.90	6678.27	802.48

**Table 2:** Household water consumption in Rybnik and surrounding areas (descriptive statistics).

	Descriptive Statistics—Water Consumption (in m³)					
	Mean ± Stand. Dev.	Median (Q25–Q75)	Min.–Max.	Confidence Interval		Stand. Error
				−95.00%	+95.00%	
Residential buildings (in thousands)	221.73 ± 22.1	213.46 (202.51–238.62)	196.46–271.92	210.37	233.09	5.36
Collective housing buildings (in thousands)	133.01 ± 5.07	131.9 (130.56–134.92)	123.7–144	130.40	135.61	1.23
Dwellings (in thousands)	15.09 ± 0.57	14.97 (14.82–15.21)	14.45–16.57	14.80	15.38	0.14
Households (in thousands)	369.82 ± 22.97	365.77 (350.58–388.69)	334.92–417.28	358.02	381.63	5.57

**Table 3:** Water consumption in the business sector in and around Rybnik (descriptive statistics).

	Descriptive Statistics—Water Consumption (in m^3^)					
	Mean ± Stand. Dev.	Median (Q25–Q75)	Min.–Max.	Confidence Interval		Stand. Error
				−95.00%	+95.00%	
Industry (in thousands)	52.29 ± 6.24	51.29 (48.87–55.09)	44.64–71.18	49.08	55.50	1.51
Trade (in thousands)	10.11 ± 1.15	9.9 (9.63–10.81)	8.13–12.45	9.52	10.71	0.28
Services (in thousands)	14.37 ± 3.55	13.59 (11.74–15.69)	10.58–22.08	12.55	16.20	0.86
Business sector (in thousands)	76.77 ± 10.36	74.15 (70.09–79.92)	64.39–105.54	71.45	82.10	2.51

**Table 4:** Water consumption in government institutions (descriptive statistics).

	Descriptive Statistics—Water Consumption (in m^3^)					
	Mean ± Stand. Dev.	Median (Q25–Q75)	Min.–Max.	Confidence Interval		Stand. Error
				−95.00%	+95.00%	
Schools (in thousands)	2.97 ± 1.34	3.19 (1.86–3.59)	1.04–5.44	2.28	3.66	0.32
Kindergartens (in thousands)	1.3 ± 0.64	1.45 (0.72–1.91)	0.13–2.02	0.97	1.62	0.16
Other government and local government institutions (in thousands)	6.25 ± 1.79	6.15 (4.7–7.11)	4.54–10.45	5.33	7.17	0.43
Government and local government institutions (in thousands)	10.52 ± 2.74	9.58 (9.01–11.61)	5.84–16.88	9.11	11.93	0.67

**Table 5:** Correlations between COVID-19 outbreak data and water consumption in households, the business sector, and government and local government institutions in and around Rybnik.

Variables	Pearson’s Linear Correlation Results		
	New Cases	Deaths	Quarantine
Residential buildings	**r = −0.592;** ***p* < 0.05**	**r = −0.534;** ***p* < 0.05**	**r = −0.531;** ***p* < 0.05**
Collective housing buildings	r = 22120.025; *p* = 0.923	r = 0.061; *p* = 0.817	r = 0.028; *p* = 0.916
Dwellings	r = 0.026; *p* = 0.921	r = 0.174; *p* = 0.505	r = 0.062; *p* = 0.814
Households	**r = −0.574;** ***p* < 0.05**	**r = −0.496;** ***p* < 0.05**	**r = −0.503;** ***p* < 0.05**
Industry	r = −0.201; *p* = 0.439	r = −0.283; *p* = 0.271	r = −0.097; *p* = 0.71
Trade	r = −0.351; *p* = 0.167	r = −0.403; *p* = 0.109	r = −0.368; *p* = 0.146
Services	**r = −0.525;** ***p* < 0.05**	**r = −0.59;** ***p* < 0.05**	**r = −0.5;** ***p* < 0.05**
Business sector	r = −0.341; *p* = 0.181	r = −0.418; *p* < 0.095	r = −0.271; *p* = 0.292
Schools	r = 0.182; *p* = 0.483	r = 0.088; *p* = 0.737	r = 0.169; *p* = 0.518
Kindergartens	r = 0.435; *p* < 0.081	r = 0.427; *p* < 0.087	r = 0.369; *p* = 0.145
Other government and local government institutions	r = −0.436; *p* < 0.08	**r = −0.498;** ***p* < 0.05**	r = −0.394; *p* = 0.118
Government and local government institutions	r = −0.094; *p* = 0.719	r = −0.182; *p* = 0.485	r = −0.089; *p* = 0.735

Statistically significant results are in bold.

**Table 6:** Values of individual pathways for the association of new cases of SARS-CoV-2 viral infection in Poland with the choice of residents of Rybnik and its surroundings to stay in households–mediating role of the functioning of the service industry in Rybnik and its surrounding areas.

Variables		Standardized Coefficient		Non-Standardized Coefficient.		T	*p*	Test F	R^2^_corr._
		Β	Stand. Error	B	Stand. Error				
Residential buildings	New cases (c)	−0.59	0.21	−0.06	0.02	−2.84	*p* < 0.05	F(1.15) = 8.08; *p* < 0.05	0.31
	New cases (c′)	−0.28	0.20	−0.03	0.02	−1.41	*p* = 0.179	F(2.14) = 10.85; *p* < 0.01	0.55
	Services (b)	0.60	0.20	3.71	1.22	3.03	*p* < 0.01		
Households	New cases (c)	−0.57	0.21	−0.06	0.02	−2.72	*p* < 0.05	F(1.15) = 7.37; *p* < 0.05	0.28
	New cases (c′)	−0.35	0.23	−0.04	0.02	−1.51	*p* = 0.153	F(2.14) = 6.09; *p* < 0.05	0.39
	Services (b)	0.43	0.23	2.80	1.48	1.88	*p* < 0.08		
Services	New cases (a)	−0.53	0.22	−0.01	0.00	−2.39	*p* < 0.05	F(1.15) = 5.71; *p* < 0.05	0.23

a—direct relationship between the independent variable and the mediator (path a), b—direct relationship between the mediator and the dependent variable (path b), c—direct relationship between the independent variable and the dependent variable (path c), c′—the relationship between the independent variable and the dependent variable with the mediator included in the model (path c′).

**Table 7:** Values of individual paths for the association of deaths due to SARS-CoV-2 viral infection in Poland with the stay of residents of Rybnik and its surrounding areas in households—mediating role of the functioning of the service industry in Rybnik and its surrounding areas.

Variables		Standardized Coefficient		Non–Standardized Coefficient		t	*p*	Test F	R^2^_corr._
		β	Stand. Error	B	Stand. Error				
Residential buildings	Deaths (c)	−0.53	0.22	0.00	0.00	−2.45	*p* < 0.05	F(1.15) = 5.98; *p* < 0.05	0.24
	Deaths (c′)	−0.15	0.22	0.00	0.00	−0.67	*p* = 0.511	F(2.14) = 9.13; *p* < 0.01	0.50
	Services (b)	0.66	0.22	4.08	1.36	3.01	p < 0.01		
Households	Deaths (c)	−0.50	0.22	0.00	0.00	−2.21	*p* < 0.05	F(1.15) = 4.89; *p* < 0.05	0.20
	Deaths (c′)	−0.20	0.26	0.00	0.00	−0.80	*p* = 0.436	F(2.14) = 4.77; *p* < 0.05	0.32
	Services (b)	0.49	0.26	3.20	1.65	1.94	*p* < 0.073		
Services	Deaths (a)	−0.59	0.21	0.00	0.00	−2.83	*p* < 0.05	F(1.15) = 8; *p* < 0.05	0.30

a—direct relationship between the independent variable and the mediator (path a), b—direct relationship between the mediator and the dependent variable (path b), c—direct relationship between the independent variable and the dependent variable (path c), c′—the relationship between the independent variable and the dependent variable with the mediator included in the model (path c′).

**Table 8:** Values of individual paths for the relationship of the number of people quarantined due to the COVID-19 epidemic in Poland with the choice of residents of Rybnik and the surrounding areas to stay in households—the mediating role of the functioning of the service industry in Rybnik and the surrounding areas.

Variables		Standardized Coefficient		Non–Standardized Coefficient		t	*p*	Test F	R^2^_corr._
		β	Stand. Error	B	Stand. Error				
Residential buildings	Quarantine (c)	−0.53	0.22	0.00	0.00	−2.43	*p* < 0.05	F(1.15) = 5.89; *p* < 0.05	0.23
	Quarantine (c′)	−0.21	0.20	0.00	0.00	−1.07	*p* = 0.302	F(2.14) = 9.9; *p* < 0.01	0.53
	Services (b)	0.64	0.20	3.96	1.24	3.20	*p* < 0.01		
Households	Quarantine (c)	−0.50	0.22	0.00	0.00	−2.26	*p* < 0.05	F(1.15) = 5.09; *p* < 0.05	0.20
	Quarantine (c′)	−0.26	0.23	0.00	0.00	−1.12	*p* = 0.282	F(2.14) = 5.27; *p* < 0.05	0.35
	Services (b)	0.48	0.23	3.13	1.51	2.08	*p* < 0.057		
Services	Quarantine (a)	−0.50	0.22	0.00	0.00	−2.24	*p* < 0.05	F(1.15) = 5.01; *p* < 0.05	0.20

a—direct relationship between the independent variable and the mediator (path a), b—direct relationship between the mediator and the dependent variable (path b), c—direct relationship between the independent variable and the dependent variable (path c), c′—the relationship between the independent variable and the dependent variable with the mediator included in the model (path c′).

**Table 9:** Values of individual paths for the association of new cases of SARS-CoV-2 viral infection in Poland with the functioning of the service industry in Rybnik and its surrounding areas—the mediating role of the functioning of other government and local government institutions in Rybnik and its surrounding areas.

Variables		Standardized Coefficient		Non–Standardized Coefficient		t	*p*	Test F	R^2^_corr._
		β	Stand. Error	B	Stand. Error				
Services	New cases (c)	−0.53	0.22	−0.01	0.00	−2.39	*p* < 0.05	F(1.15) = 5.71; *p* < 0.05	0.23
	New cases (c′)	−0.13	0.07	0.00	0.00	−1.91	*p* < 0.076	F(2.14) = 133.58; *p* < 0.001	0.94
	Other government and local government institutions (b)	0.91	0.07	1.81	0.13	13.77	*p* < 0.001		
Other government and local government institutions	New cases (a)	−0.44	0.23	0.00	0.00	−1.88	*p* < 0.08	F(1.15) = 3.53; *p* < 0.08	0.14

a—direct relationship between the independent variable and the mediator (path a), b—direct relationship between the mediator and the dependent variable (path b), c—direct relationship between the independent variable and the dependent variable (path c), c′—the relationship between the independent variable and the dependent variable with the mediator included in the model (path c′).

**Table 10:** Values of individual paths for the association of deaths caused by SARS-CoV-2 infection in Poland with the functioning of the service industry in Rybnik and the surrounding areas—the mediating role of the functioning of other government and local government institutions in Rybnik and the surrounding areas.

Variables		Standardized Coefficient		Non–Standardized Coefficient		t	*p*	Test F	R^2^_corr._
		β	Stand. Error	B	Stand. Error				
Services	Deaths (c)	−0.59	0.21	0.00	0.00	−2.83	*p* < 0.05	F(1.15) = 8; *p* < 0.05	0.30
	Deaths (c′)	−0.14	0.07	0.00	0.00	−2.14	*p* < 0.05	F(2.14) = 141.04; *p* < 0.001	0.95
	Other government and local government institutions (b)	0.90	0.07	1.78	0.13	13.38	*p* < 0.001		
Other government and local government institutions	Deaths (a)	−0.50	0.22	0.00	0.00	−2.22	*p* < 0.05	F(1.15) = 4.93; *p* < 0.05	0.20

a—direct relationship between the independent variable and the mediator (path a), b—direct relationship between the mediator and the dependent variable (path b), c—direct relationship between the independent variable and the dependent variable (path c), c′—the relationship between the independent variable and the dependent variable with the mediator included in the model (path c′).

**Table 11:** Values of individual paths for the relationship between the number of people quarantined due to the COVID-19 epidemic in Poland and the functioning of the service industry in Rybnik and its surrounding areas—the mediating role of the functioning of other government and local government institutions in Rybnik and its surrounding areas.

Variables		Standardized Coefficient		Non–Standardized Coefficient		t	*p*	Test F	R^2^_corr._
		Β	Stand. Error	B	Stand. Error				
Services	Quarantine (c)	–0.50	0.22	0.00	0.00	−2.24	*p* < 0.05	F(1.15) = 5.01; *p* < 0.05	0.20
	Quarantine (c′)	−0.14	0.06	0.00	0.00	−2.25	*p* < 0.05	F(2.14) = 144.87; *p* < 0.001	0.95
	Other government and local government institutions (b)	0.91	0.06	1.81	0.12	14.62	*p* < 0.001		
Other government and local government institutions	Quarantine (a)	−0.39	0.24	0.00	0.00	−1.66	*p* = 0.118	F(1.15) = 2.76; *p* = 0.118	0.10

a—direct relationship between the independent variable and the mediator (path a), b—direct relationship between the mediator and the dependent variable (path b), c—direct relationship between the independent variable and the dependent variable (path c), c′—the relationship between the independent variable and the dependent variable with the mediator included in the model (path c′).

**Table 12:** Summary of recorded total and partial mediations in the context of the functioning of the residents of Rybnik and service companies and public institutions in this city in the period of the most significant threat of the SARS-CoV-2 virus.

Independent Variable	Dependent Variable	Mediator	Pathways c/c′	Mediation
New cases	Residential buildings	Services	β = −0.59; *p* < 0.05/ β = −0.28; *p* = 0.179	Total
Deaths	Residential buildings	Services	β = −0.53; *p* < 0.05/ β = −0.15; *p* = 0.511	Total
Quarantine	Residential buildings	Services	β = −0.53; *p* < 0.05/ β = −0.21; *p* = 0.302	Total
Deaths	Services	Other government and local government institutions	β = −0.59; *p* < 0.05/ β = −0.14; *p* < 0.05	Partial

## Data Availability

Data are contained within the article.
